# *miR-141* regulates TGF-β1-induced epithelial-mesenchymal transition through repression of *HIPK2* expression in renal tubular epithelial cells

**DOI:** 10.3892/ijmm.2014.2008

**Published:** 2014-11-24

**Authors:** YUANHANG HUANG, JUNRONG TONG, FENG HE, XINPEI YU, LIMING FAN, JING HU, JIANGPING TAN, ZHENGLIANG CHEN

**Affiliations:** 1 Department of Immunology, School of Basic Medical Sciences, Southern Medical University, Guangzhou, Guangdong 510515, P.R. China; 2 Department of Nephrology, School of Basic Medical Sciences, Southern Medical University, Guangzhou, Guangdong 510515, P.R. China; 3 Geriatric Infection and Organ Function Support Laboratory, Guanzhou General Hospital of Guanzhou Military Command, Guangzhou, Guangdong 510010, P.R. China

**Keywords:** microRNA, *miR-141*, epithelial mesenchymal transition, renal tubulointerstitial fibrosis, TGF-β1, FSP1, HIPK2

## Abstract

Epithelial-mesenchymal transition (EMT) plays a critical role in embryonic development, wound healing, tissue regeneration, cancer progression and organ fibrosis. The proximal tubular epithelial cells undergo EMT, resulting in matrix-producing fibroblasts and thereby contribute to the pathogenesis of renal fibrosis. The profibrotic cytokine, TGF-β, is now recognized as the main pathogenic driver that has been shown to induce EMT in tubular epithelial cells. Increasing evidence indicate that HIPK2 dysfunction may play a role in fibroblasts behavior, and therefore, HIPK2 may be considered as a novel potential target for anti-fibrosis therapy. Recently, members of the *miR-200* family (*miR-200a*, *b* and *c* and *miR-141*) have been shown to inhibit EMT. However, the steps of the multifactorial renal fibrosis progression that these miRNAs regulate, particularly *miR-141*, are unclear. To study the functional importance of *miR-141* in EMT, a well-established *in vitro* EMT assay was used to demonstrate renal tubulointerstitial fibrosis; transforming growth factor-*β*1-induced EMT in HK-2 cells. Overexpression of *miR-141* in HK-2 cells, either with or without TGF-β1 treatment, hindered EMT by enhancing E-cadherin and decreasing vimentin and fibroblast-specific protein 1 expression. *miR-141* expression was repressed during EMT in a dose- and time-dependent manner through upregulation of *HIPK2* expression. Ectopic expression of *HIPK2* promoted EMT by decreasing E-cadherin. Furthermore, co-transfection of *miR-141* with the *HIPK2* ORF clone partially inhibited EMT by restoring E-cadherin expression. *miR-141* downregulated the expression of *HIPK2* via direct interaction with the 3′-untranslated region of *HIPK2*. Taken together, these findings aid in the understanding of the role and mechanism of *miR-141* in regulating renal fibrosis via the TGF-β1/*miR-141*/HIPK2/EMT axis, and *miR-141* may represent novel biomarkers and therapeutic targets in the treatment of renal fibrosis.

## Introduction

Renal fibrosis is the frequent final outcome of a wide variety of progressive chronic kidney diseases (1). Epithelial-mesenchymal transition (EMT) is a central mechanism in tubulointerstitial fibrosis, in which tubular epithelial cell loss is accompanied by the deposition of extracellular matrix (ECM) and accumulation of fibroblasts and inflammatory cells in the intersitium (2–5). The phenotypic conversion of epithelial cells to myofibroblast (with expression of vimentin and less expression of E-cadherin) is the main feature of this process (6). Increasing evidence suggests that numerous genes are involved in tubular EMT (7–10). The transforming growth factor (TGF)-β/Smad pathway is a key promoter of this process (11,12). Increased glomerular expression of TGF-β has been reported in experimental and human kidney disease (1,5,13). Mice with increased plasma TGF-β1 levels exhibited enhanced renal fibrosis (14). Through the induction of target genes, TGF-β signaling promotes fibroblast survival and proliferation. The range of TGF-β-target genes include microRNAs (miRNAs or miRs) (15).

miRNAs are small (21–23-nt) non-coding RNA molecules that regulate gene expression by interacting with multiple mRNAs and inducing translational suppression or degradation of mRNA (16). miRNAs are involved in regulating diverse physiological processes ranging from embryogenesis, organ development, oncogenesis and the initial step in EMT (17–19). Three miRNA families, *miR-21*, *miR-200* and *miR-29*, are regulated by TGF-β and have been shown to modulate renal fibrosis either by amplifying TGF-β signaling and promoting fibrosis (*miR-21*) or by inhibiting EMT and reducing fibrosis (*miR-29* and *miR-200*) (20–22). Five members of the *miR-200* family identified thus far are *miR-200a*, *miR-200b*, *miR-200c*, *miR-429* and *miR-141*. Published data suggest that the *miR-200* family inhibit EMT through directly targeting zinc finger E-box-binding homeobox (*ZEB*)*-1* and *ZEB-2*, which are E-cadherin transcriptional repressors in kidney tubular cells (23). Therefore, approaches to correct miRNA expression represent the novel therapeutic strategies for these diseases.

Homeodomain interacting protein kinase 2 (HIPK2) is a member of an evolutionary conserved family of serine/threonine kinases (24), which is considered as a tumor suppressor gene and mediates the activation of Wnt, Notch, and TGF-β-induced signaling (25–27). HIPK2 is also considered as a co-regulator of an increasing number of transcription factors modulating numerous different basic cellular processes, including apoptosis, proliferation, differentiation and development (24,28–30). Recently, HIPK2 has also been identified as a key regulator in idiopathic pulmonary fibrosis (IPF) and kidney fibrosis (31,32). In the kidney, HIPK2 mediates apoptosis and EMT of renal tubular epithelial cells, contributing to fibrosis. HIPK2 may be a potential target for anti-fibrosis therapy (31). Given the ability of the *miR-200* family to inhibit EMT and the evidence of HIPK2 in tissue fibrosis, whether *miR-141* ameliorates tubulointerstitial fibrosis was investigated by inhibition of EMT through targeting HIPK2. In the present study, the first aim was to define a role of *miR-141* in regulating EMT in TGF-β-treated human kidney 2 (HK-2) cells (normal renal tubular epithelial cells). *miR-141* hindered EMT by upregulating E-cadherin and downregulating vimentin and fibroblast-specific protein 1 (FSP1) expression through direct targeting of HIPK2, by binding to its three prime untranslated region (3′-UTR).

## Materials and methods

### Cell lines and transfection

HK-2 cell lines were purchased from American Type Culture Collection (Manassas, VA, USA) and maintained in RPMI-1640 medium (Invitrogen, Carlsbad, CA, USA) supplemented with 10% fetal bovine serum (Gibco, Carlsbad, CA, USA), 100 U/ml penicillin and 100 *μ*g/ml streptomycin in a humidified 5% CO_2_ incubator at 37°C. Ectopic expression of *miR-141* in HK-2 cells was achieved by transfection with *miR-141* mimics (Genepharma, Shanghai, China) using Lipofectamine 2000 (Invitrogen). Overexpression of *HIPK2* was performed using the *HIPK2* ORF expression clone (GeneCopoecia, Guangzhou, China).

### RNA extraction and SYBR green quantitative polymerase chain reaction (qPCR)

Total RNA was extracted using the Trizol reagent as recommended by the manufacturer (Invitrogen). RNA quality and concentration were evaluated by spectrophotometry using a NanoDrop 2000c instrument (Thermo Scientific, Rockford, IL, USA). For miRNA analysis, mature *miR-141* was detected using a Hairpin-it™ miRNAs qPCR Quantitation kit (GenePharma, Shanghai, China). *U6* served as an internal reference. For *HIPK2* mRNA analysis, qPCR was performed using the Power SYBR-Green PCR master mix (Takara, Dalian, China) on an ABI 7900HT PCR machine (Applied Biosystems, Foster City, CA, USA), and data were normalized to *β*-actin and further normalized to the negative control, unless otherwise indicated. Data analysis was performed using the 2^−ΔΔCt^ method. Human recombinant TGF-β1 was purchased from Cell Signaling Technology (no. 8915; Danvers, MA, USA), reconstituted in 20 mM citrate (pH 3.0) at a concentration of 100 *μ*g/ml. Further dilution was made in PBS containing 2 mg/ml albumin, stored at −20°C for future use.

### Immunoblot analysis

Whole cell lysates were collected in radioimmunoprecipitation assay buffer with protease inhibitor cocktail (Roche, Indianapolis, IN, USA). Protein concentration was measured using a bicinchoninic acid protein assay kit (Thermo Scientific). Reduced protein (10–30 *μ*g) in Laemmli sample buffer was resolved using 6–12% sodium dodecyl sulfate polyacrylamide gel and transferred to a nitrocellulose membrane (Bio-Rad, Hercules, CA, USA). Membranes were blocked with 5% skimmed dry milk (Bio-Rad) in Tris-buffered saline (TBS) with 0.05% Tween 20 (TBST) buffer for 1 h at room temperature, incubated with primary antibody overnight at 4°C, followed by the appropriate secondary immunoglobulin G antibody; anti-mouse, rabbit-horseradish peroxidase (Bio-Rad). Membranes were washed thoroughly between steps using TBST, and developed using the ECL Western blotting detection kit (Bio-Rad). All the primary antibodies used were from Cell Signaling Technology. The antibodies used were the following: HIPK2 (no. 5091), vimentin (no. 5741), FSP1 (no. 13018), E-cadherin (no. 3195) and β-actin (no. 4967). The blots were stripped using stripping buffer (Thermo Scientific) prior to reprobing. *β*-actin was used as an endogenous protein for normalization. Images were analyzed by Quantity One software (Bio-Rad).

### HIPK2 3′-UTR luciferase reporter assays

The 3′-UTR for *HIPK2* was PCR-amplified from genomic DNA extracted from HK-2 cells. The PCR primers used to amplify the *Hipk2* 3′-UTR were forward, 5′-CTCGAGACACTTTGCATAACGTATA-3′; and reverse, 5′-GCGGCCGCATTGGAACAGTAGTCATAT-3′, whereas the primers used to amplify the mutant *Hipk2* 3′-UTR (with a mutant seed sequence of miR-141) were forward, 5′-TTCAAATGAATATTTCGTCTAAATAAATAAAG-3′; and reverse, 5′-TGTAGACGAAATATTCATTTGAATTTTCAAG-3′. The mutant *Hipk2* 3′-UTR was identical to the wild-type sequence except for the seed regions, in which the complementary sequence was used. The mutant version was generated using the Stratagene QuikChange™ site-directed mutagenesis strategy (Stratagene, La Jolla, CA, USA). Amplified 3′-UTRs were cloned downstream of the luciferase coding region in the psiCECK™-2 vector (Promega, Madison, WI, USA). The fidelity of all the constructs utilized in the study was confirmed by sequencing (ABI PRISM 377 automated sequencer; Southern Medical University, Guangzhou, Guangdong, China) and subsequent sequence alignment (NCBI BLAST) using GenBank™ accession gene ID 28996. To assess the effect of *miR-141* on reporter activity, HK-2 cells were seeded in 96-well clusters 24 h prior to transfection. The following day, medium was replaced with OptiMEM (Invitrogen), and cells were co-transfected with 100 ng reporter plasmids along with 100 ng control *Rnilla*-luciferase plasmid and either scrambled control miRNA (Vector) or *miR141* (*miR-141* Sponge) using Lipofectamine 2000 (Invitrogen). The cells were collected 48 h post-transfection and luciferase activity was detected using a dual-luciferase reporter assay system (Promega). All the experiments were performed in triplicate, and each experiment was repeated at least three times.

### Statistical analysis

Values are shown as mean ± standard error, unless otherwise specified. SPSS 17.0 (Pearson; SPSS, Inc., Chicago, IL, USA) was used to analyze data by unpaired student *t* test or by analysis of variance. P<0.05 was considered to indicate a statistically significant difference.

## Results

### miR-141 downregulates the expression of EMT markers

**Figure 1:**
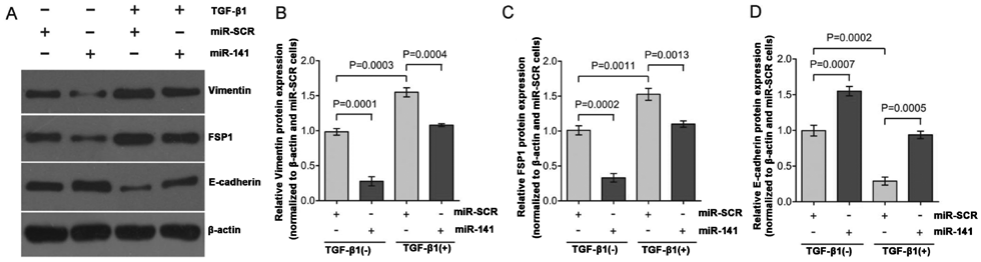
Overexpression of *miR-141* inhibits EMT by downregulation of vimentin and FSP1 and upregulation of E-cadherin. (A) HK-2 cells were transfected with negative scrambled control miRNA (miR-SCR) or with *miR-141*, and were treated with or without TGF-β1 (2 ng/ml) for 48 h. The cell lysates were collected and immunoblotted with vimentin, FSP1 and E-cadherin antibodies as indicated. The figure is a representative of three experiments with similar results. (B–D) The western blotting results in A were quantified and shown in a graph format. The expression of the EMT markers (vimentin, FSP1 and E-cadherin) was normalized to β-actin, and the expression for the different groups was determined as a relative change from miR-SCR in the absence of TGF-β1 treatment and shown as mean ± standard error. EMT, epithelial-mesenchymal transition; FSP1, fibroblast-specific protein 1; TGF, transforming growth factor.

**Figure 2:**
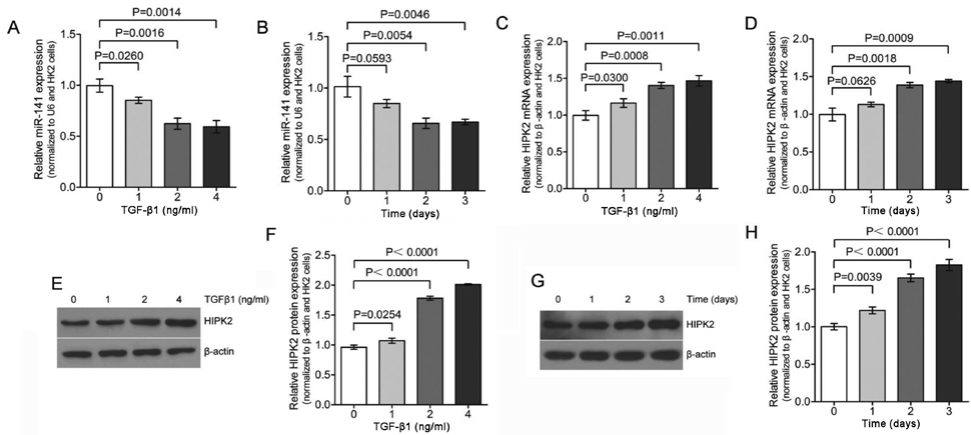
TGF-β1 induces downregulation of *miR-141* accompanied by up regulation of *HIPK2*. HK-2 cells were treated with (A) TGF-β1 (0, 1, 2 and 4 ng/ml for 2 days) or (B) TGF-β1 (2 ng/ml) for 0–3 days. The expression of *miR-141* was assessed by qPCR and normalized to *U6* expression. The expression for the various dose points was determined as a relative change in the absence of TGF-β1 treatment and shown as mean ± standard error (S.E). HK-2 cells were incubated with (C and E) TGF-β1 (0, 1, 2 and 4 ng/ml for 2 days) or (D and G) TGF-β1 (2 ng/ml) for 0–3 days and the *HIPK2* expression level was assessed by (C and D) qPCR and (E and G) western blotting, which were consistent with the RNA expression analysis in C and D, respectively. (F and H) The results from the western analysis in E and G were quantified and subjected to densitometry and shown as a graph (P-values are compared to the control). The relative expression of HIPK2 was normalized to β-actin expression and the expression for the various dose points was determined as a relative change from dose 0 and shown as mean ± S.E. TGF, transforming growth factor; HIPK2, homeodomain-interacting protein kinase 2; qPCR, quantitative polymerase chain reaction.

To study EMT, TGF-β1-induced EMT was used in the HK-2 cells. Exposure of HK-2 cells to TGF-β1 (2 ng/ml) for 48 h resulted in a significant decreased expression of the epithelial marker, E-cadherin, and increased expression of mesenchymal markers, vimentin and FSP1 (Fig. 1A; lane 1 compared to lane 3; lane 2 compared to lane 4). These hallmark shifts at the molecular level indicate a successful EMT program in HK-2 cells. *miR-200* is enriched in the kidney and lung, where it functions to maintain epithelial differentiation. Among the five *miR-200* family members, *miR-200a* and *miR-141* share the same seed sequence, AACACU. Therefore, these two members may have the same mRNA targets. A recent study found that *miR-200a* significantly influenced the development and progression of TGF-β-dependent EMT and fibrosis *in vitro* and *in vivo* (33). To study the actions of *miR-141* in the regulation of EMT, HK-2 cells were transfected with *miR-141* and changes of the gene expression were compared with the negative scrambled control (miR-SCR). As shown in Fig. 1A (lane 3 verses lane 4), HK-2 cells transfected with *miR-141* responded to TGF-β1 treatment with >70% reduction of vimentin (Fig. 1B) and FSP1 expression (Fig. 1C), and >50% increase of E-cadherin expression (Fig. 1D) as compared to the control. Overall, these results indicate that *miR-141* is capable of functionally disrupting the EMT switch in HK-2 cell by maintaining relative high levels of E-cadherin expression, which is consistent with previous studies reporting that the *miR-200* family inhibits EMT by targeting E-cadherin transcriptional repressors (23,34). *TGF-β1 downregulates the expression of miR-141 accompanied by upregulation of HIPK2.* Previous published data reported that treatment with TGF-β1 lead to decreased expression of the *miR-200* family, including *miR-141* (33). Therefore, qPCR analysis was used to reveal that the expression of *miR-141* was significantly downregulated in HK-2 cell following TGF-β1 treatment in a dose- and time-dependent manner (Fig. 2A and B). The expression of *miR-141* was reduced to <30% of the pretreatment level within 48 h of TGF-β1 exposure (Fig. 2B). These results confirm that TGF-*β*1 treatment lead to decreased expression of *miR-141*.

A published systematic approach identified HIPK2 as a key regulator of renal fibrosis (31). Another recent study implicated dysregulated HIPK2 levels in idiopathic pulmonary fibrosis (IPF) (32). Therefore, the role of HIPK2 in TGF-β1 treated HK-2 cells was investigated. Initial qPCR demonstrated TGF-β1-mediated induction of HIPK2 in HK-2 kidney tubular cells (Fig. 2C and D) in a dose- and time-dependent manner, which corresponded to the decreased *miR-141* expression. To verify the induction of HIPK2, the expression was analyzed by western blotting in HK-2 cells. As shown in Fig. 2E and F, HK-2 cells exposed to different dose of TGF-β1 for 2 days increased the expression of HIPK2 significantly. The maximal *HIPK2* mRNA and protein expression levels at 4 ng/ml TGF-β1 treatment were ~1.5-2-fold higher than the control. Furthermore, HK-2 cells exposed to TGF-β1 (2 ng/ml) for 0–3 days also increased the expression of HIPK2 significantly (Fig. 2G and H). This result is consistent with the observation shown in Fig. 2C and D, which means that the changes in HIPK2 expression were also observed at the protein level. Collectively, these results indicated that lower expression levels of *miR-141* were significantly associated with higher levels of *HIPK2* mRNA and protein expression in the same set of TGF-β1 treatment HK-2 cells.

### Ectopic expression of HIPK2 promotes EMT

**Figure 3:**
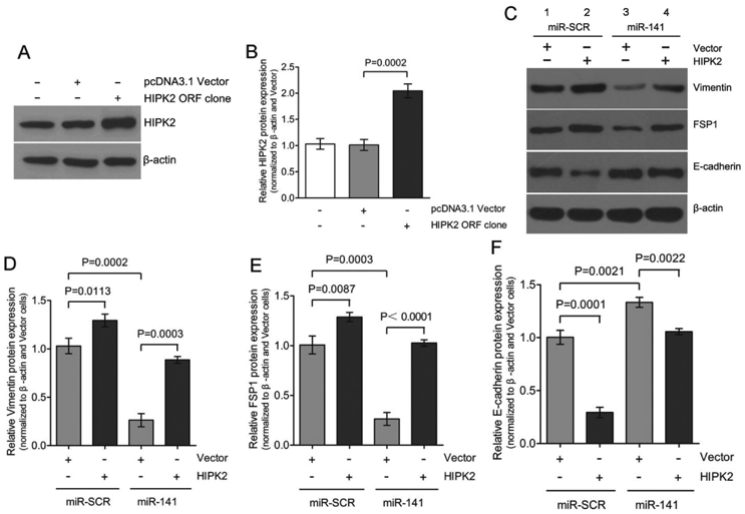
Ectopic expression of HIPK2 promotes EMT in HK-2 cells. (A) HK-2 cells were transfected with pcDNA3.1 empty vector or *HIPK2* ORF. The cell lysates were immunoblotted with HIPK2 antibody as indicated, to confirm the expression of HIPK2. (B) The western blotting results in (A) were quantified and shown in a graph format. The intensities of the bands corresponding to HIPK2 were compared to those corresponding to β-actin. (C) HK-2 cells expressing miR-SCR or *miR-141* were transfected with empty vector or 2 *μ*g *HIPK2* ORF. Post-transfection (48 h), the protein levels of the EMT markers were analyzed by immunoblotting. The figure is representative of three experiments with similar results. (D–F) The western blotting results in (C) were quantified to determine whether a statistically significant difference exists between the groups and they are shown in a graph format (P<0.05 compared to the control). The intensities of the bands corresponding to vimentin, FSP1 and E-cadherin were compared to those corresponding to β-actin. The expression of EMT was determined as a relative change from miR–SCR transfected with vector and shown as mean ± standard error. HIPK2, homeodomain-interacting protein kinase 2; EMT, epithelial-mesenchymal transition; FSP1, fibroblast-specific protein 1.

Dysregulation of HIPK2 has been implicated in increased proliferation, as it is typical in cancer or fibrosis (35,36). A recently published study identified HIPK2 as a key regulator of kidney fibrosis (31). Whether HIPK2 was sufficient to induce EMT in the absence of TGF-β1 and what the potential role of *miR-141* was during this process were investigated. Transient expression of *HIPK2* ORF in HK-2 cells resulted in a considerable upregulation of HIPK2 expression to 2.0±0.2-fold (Fig. 3A and B) accompanied by the gain of mesenchymal markers, vimentin and FSP1, and the loss expression of epithelial marker E-cadherin (Fig. 3C; lanes 1 and 2). Co-transfection of *miR-141* with the *HIPK2* ORF clone partially restored E-cadherin expression and attenuated vimentin and FSP1 induction (Fig. 3C; lanes 2 and 4). These results are consistent with our previous results that downregulation of *miR-141* is accompanied by upregulation of HIPK2 during TGF-β1-induced EMT in HK-2 cells. Therefore, downregulation of *miR-141* appears to be required for mediating the upregulation of HIPK2 in the EMT process. HIPK2 can mimic the TGF-β1-induced EMT process in HK-2 cells.

### miR-141 directly targets HIPK2

**Figure 4:**
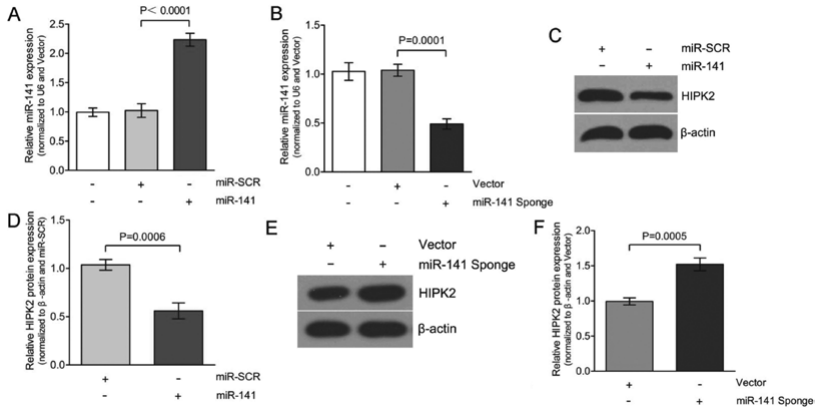
*miR-141* represses the expression of HIPK2. HK-2 cells were transfected with (A) miR-SCR or with *miR-141*, or (B) with control (Vector) or the inhibitor of *miR-141* (*miR-141* Sponge), and RNA was harvested after 2 days. The expression of *miR-141* was assessed by qPCR and normalized to *U6* expression; shown as mean ± standard error (S.E). Western blot analysis demonstrated a decrease in HIPK2 by (C) *miR-141* and (E) *miR-141* Sponge. The figures are representative of three experiments with similar results. (D and F) The western blotting results in C and E were quantified to determine whether a statistically significant difference exists between the groups and are shown in a graph format (P<0.05 compared to control). The intensities of all bands corresponding to HIPK2 were compared to those corresponding to β-actin. The expression of HIPK2 was determined as a relative change from miR-SCR or vector transfection and shown as mean ± S.E. HIPK2, homeodomain-interacting protein kinase 2; qPCR, quantitative polymerase chain reaction.

The critical role of *miR-141* in EMT prompted the identification of the genes that were directly regulated by *miR-141*. Whether overexpression of *miR-141* would alter HIPK2 protein expression was initially determined. As shown in Fig. 4A and C, transfecting with *miR-141* increased expression of *miR-141* to ~2.3-fold, compared to the transfection of miR-SCR in the HK-2 cells. This increase in *miR-141* expression decreased HIPK2 expression to an average of 50±5% in the three experiments (Fig. 4D). Based on this observation, we hypothesize that the *miR-141* inhibitor (*miR-141* Sponge) should not be able to decrease HIPK2 expression. To test this hypothesis, HK-2 cells were transfected with either vector or *miR-141* Sponge and HIPK2 protein expression was determined. The overexpression of *miR-141* Sponge caused a downregulation of the *miR-141* levels to ~0.5-fold compared to the transfection of the vector in the HK-2 cells (Fig. 4B and E). Consequently, the decreased expression of *miR-141* caused upregulation of HIPK2 expression, with an average of 1.5±0.1-fold in three experiments (Fig. 4F). Therefore, the expression of HIPK2 was significantly decreased in *miR-141*-transfected cells. Taken together, TGF-β1 (Fig. 2) and *miR-141* inhibitor (*miR-141* Sponge) caused a similar effect on HIPK2 expression in HK-2 cells.

**Figure 5:**
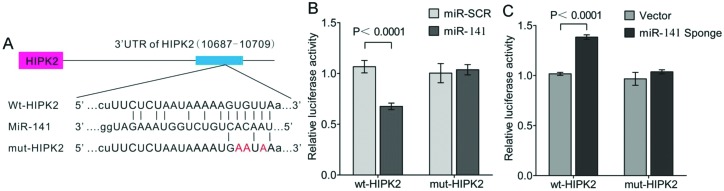
*miR-141* directly targets HIPK2 by binding to its 3′UTR. (A) Alignment of the *miR-141* sequences and the putative targeted area of the 3′-UTR of human *HIPK2* (http://www.targetscan.org). Also shown is the altered sequence of the mutant 3′-UTR of *HIPK2*. Normalized activity of luciferase reporter with the *HIPK2* 3′-UTR or mut-HIPK2 (1 *μ*g) in HK-2 cells in the presence of (B) the co-transfected negative control (miR-SCR) or *miR-141* (100 nmol/l), or (C) the co-transfected vector or *miR-141* Sponge (100 nmol/l). Luciferase activity was measured after (B) 24 and (C) 48 h. The data are the means from a representative experiment measured in triplicate and are presented as mean ± standard error, and are shown as the ratio of firefly/*Renilla* luciferase activity. HIPK2, homeodomain-interacting protein kinase 2; UTR, untranslated region.

As *miR-141* and *HIPK2* 3′-UTR share the same seed sequence as shown (Fig. 5A), whether HIPK2 is the direct target of *miR-141* on translational repression was further investigated. In these experiments, luciferase reporter constructs were used, incorporating a wild-type or mutant 3′-UTR of *HIPK2* in which the sequence corresponding to the seed region was altered. Co-transfection of wild-type *HIPK2* luciferase reporter construct or mutant 3′-UTR of *HIPK2* with *miR-141* or miR-SCR in HK-2 cells resulted in a significantly reduced *HIPK2* 3′-UTR luciferase activity expression. This demonstrated that *miR-141* directly repressed luciferase activity with the wild-type 3′-UTR of *HIPK2* (Fig. 5B), but not with the mutant 3′-UTR. By contrast, *miR-141* Sponge increased luciferase activity with the wild-type *HIPK2*, but not with the mutant version of *HIPK2* (Fig. 5C).

## Discussion

Tubular EMT is a series of highly-regulated pathological events, which is one of the major mechanisms leading to renal fibrosis (1,5,13,37). miRNAs are recognized to be critical regulators of a number of important developmental, homeostatic and pathogenic pathways. Recent findings suggest that miRNAs are essential for kidney development and homeostasis. The *miR-200* family members were clearly downregulated in cells that had undergone EMT in response to TGF-β, and the expression of the *miR-200* family alone was sufficient to prevent TGF-β-induced EMT (38). The study by Tamagawa *et al* (34) demonstrated the role of *miR-200c*/*miR-141* in the regulation of EMT and migration in head and neck squamous cell carcinoma. However, the functional involvement of *miR-141* in EMT associated with tubulointerstitial fibrosis, as well as direct targeting by *miR-141*, has not been investigated. In the present study, *miR-141* influenced the progression of TGF-β1-induced EMT *in vitro*. An EMT model was initially established using TGF-β1-treated HK-2 cells by observing upregulation of vimentin and FSP1 and downregulation of E-cadherin (Fig. 1A; lanes 1 and 3) at the protein levels. Overexpression of *miR-141* inhibited EMT progression in TGF-β1-treated HK-2 cells by maintaining high E-cadherin expression levels and low vimentin and FSP1 expression levels (Fig. 1A; lanes 3 and 4). Furthermore, *miR-141* was found to be downregulated during TGF-β1-induced EMT in HK-2 cells (Fig. 2). Given that reduced *miR-141* levels are associated with increased EMT expression, and that restoring expression of *miR-141* prevents EMT in HK-2 cells, *miR-141* appears to play an important role in EMT, and therefore, renal fibrosis. Wang *et al* (33) also found that in early and more advanced models of kidney disease, the downregulation of *miR-141* was also associated with increased TGF-β expression and renal scarring. Additionally, downregulated expression of *miR-141* was accompanied by upregulated *HIPK2* expression (Fig. 2). These observations suggest a possible regulatory network that drives the increased expression of HIPK2 during TGF-β1-induced EMT, perspectives that warrant further investigation.

Although a number of different factors contribute to renal fibrosis, the most known and studied profibrotic agent is TGF-β1, which is increased in the diabetic kidney (39). Recently, HIPK2 has been also identified as a main regulator of kidney fibrosis (31). In the kidney, HIPK2 mediates apoptosis and EMT of renal tubular epithelial cells, contributing to fibrosis (31). In the present study, overexpression of *HIPK2* resulted in the upregulation of vimentin and FSP1, and downregulation of E-cadherin (Fig. 3), which indicates that HIPK2 mimicked TGF-β1-induced EMT in HK-2 cells. These initial studies suggested that overexpression of *miR-141* inhibited TGF-β1-induced EMT (Fig. 1). Given the possible link between *miR-141* and HIPK2, whether overexpression of *miR-141* hindered HIPK2 expression in HK-2 cells was investigated further. To test this hypothesis, miR-SCR or *miR-141* were transfected with either vector or the *HIPK2* ORF clone and the EMT markers expression was determined. The ability of *miR-141* to attenuate HIPK2 expression has potential implications and may prove to be an attractive option for targeting the EMT pathway.

Certain miRs are well known to be indiscriminate and may bind to the UTR regions of a number of different genes. The results of the present study showed that overexpression of *miR-141* significantly reduced the expression of HIPK2 (Fig. 4), and therefore, it is possible that the 3′-UTR of HIPK2 may harbor a target site for *miR-141*. The TargetScan database indicated that HIPK2 had a target site for *miR-141*, as shown in Fig. 5A. In the study, *miR-141* was shown to be a direct repressor of HIPK2 as it targeted the 3′-UTR of this gene, as shown by experiments using the wild-type and mutant HIPK2 3′-UTR-luciferase constructs. HIPK2 was a transcriptional cofactor in the downstream TGF-β/BMP signaling pathway (27,31,40–42). Notably, loss of HIPK2 reduced cellular responses to TGF-β during neuronal development and in mouse models of renal fibrosis (27,40). In addition, the present experimental results confirmed that HIPK2 was a functional target of *miR-141* in HK-2 cells. There are several lines of evidence to support this. Firstly, protein expression of HIPK2 was significantly decreased in the *miR-141* group compared to the miR-SCR group (Fig. 4). Overexpression of *miR-141* significantly downregulated HIPK2 by directly targeting the 3′UTR of *HIPK2* mRNA, confirmed using the luciferase reporter-gene assays (Fig. 5). This effect was largely eliminated when the sites in *HIPK2* 3′UTR targeted by *miR-141* were mutated. Overexpression of *HIPK2* mimicked TGF-β1-induced EMT, and overexpression of *miR-141* rescued the expression of epithelial marker E-cadherin and inhibited HIPK2-induced EMT (Fig. 3C and F; lanes 3 and 4). These results strongly suggested that *miR-141*, through directly targeting of *HIPK2*, plays an essential role in renal proximal tubular EMT. *miR-141* suppresses the expression of *HIPK2* through directly interacting with its wild-type 3′-UTR.

In the present study, ectopic expression of *miR-141* hindered the progression of EMT in TGF-β1-treated HK-2 cells by downregulation of HIPK2 expression, maintaining a high expression level of E-cadherin. Since HIPK2 has been indicated to be involved in the progression of EMT by suppression of E-cadherin expression, it may provide important targets for therapeutic strategies. The findings in the study suggested that *miR-141* was downregulated in TGF-β1-treated HK-2 cells. This not only reveals a close association between the *miR-141* expression level and epithelial phenotypic conversion, but also implicates a potential role of *miR-141* in the regulation of tubular dedifferentiation, which is a frequent pathogenesis in tubular interstitial fibrosis in a number of types of chronic kidney disease. Evidently, more studies are required to further characterize the role of *miR-141* in the pathogenesis of EMT, as well as to depict detailed molecular pathways that are involved in EMT and kidney fibrosis following chronic renal injury.

**Figure 6:**
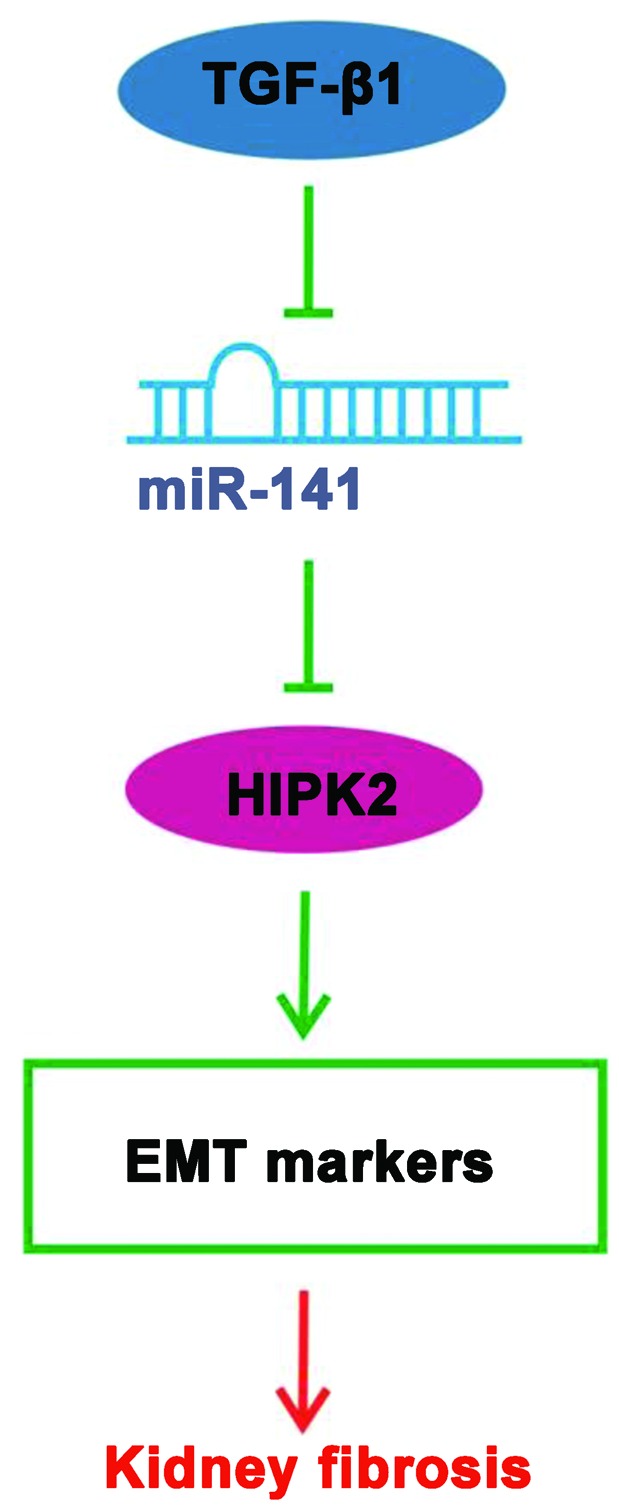
Schematic model depicting that *miR-141* controls EMT markers via repression of *HIPK2* expression in HK-2 cells based on the data of the present study. TGF-β1 downregulated the expression of *miR-141*, upregulated the expression of *HIPK2*, and consequently regulated the EMT markers in HK-2 cells. Simultaneously, *miR-141* targeted *HIPK2* to regulate the protein expression of the EMT markers (vimentin, FSP1 and E-cadherin). The process plays an important role in the formation of renal fibrosis. EMT, epithelial-mesenchymal transition; HIPK2, homeodomain-interacting protein kinase 2; TGF, transforming growth factor; FSP1, fibroblast-specific protein 1.

In conclusion, the present results indicate that *miR-141* coordinately regulates the TGF-β1-induced EMT process through the HIPK2 signaling pathway (Fig. 6) and exogenous overexpression of *miR-141* may represent a promising approach for targeted kidney fibriosis therapies.

## Abbreviations

EMT: epithelial mesenchymtion transition
HIPK2: homeodomain-interacting protein kinase 2
miRNA: microRNA
TGF-β: transforming growth factor-β
HK-2: human kidney 2
FSP1: fibroblast-specific protein 1
UTR: untranslated region
qPCR: quantitative polymerase chain reaction
ORF: open reading frame

## References

[1] Liu Y (2004). Epithelial to mesenchymal transition in renal fibrogenesis: pathologic significance, molecular mechanism, and therapeutic intervention. J Am Soc Nephrol.

[2] Zeisberg M, Kalluri R (2004). The role of epithelial-to-mesenchymal transition in renal fibrosis. J Mol Med (Berl).

[3] Kalluri R, Weinberg RA (2009). The basics of epithelial-mesenchymal transition. J Clin Invest.

[4] Strutz F, Okada H, Lo CW, Danoff T, Carone RL, Tomaszewski JE, Neilson EG (1995). Identification and characterization of a fibroblast marker: FSP1. J Cell Biol.

[5] Strutz F, Müller GA (2006). Renal fibrosis and the origin of the renal fibroblast. Nephrol Dial Transplant.

[6] Liu Y (2010). New insights into epithelial-mesenchymal transition in kidney fibrosis. J Am Soc Nephrol.

[7] Li Y, Yang J, Luo JH, Dedhar S, Liu Y (2007). Tubular epithelial cell dedifferentiation is driven by the helix-loop-helix transcriptional inhibitor id1. J Am Soc Nephrol.

[8] Tan R, Zhang J, Tan X, Zhang X, Yang J, Liu Y (2006). Downregulation of SnoN expression in obstructive nephropathy is mediated by an enhanced ubiquitin-dependent degradation. J Am Soc Nephrol.

[9] Yang J, Shultz RW, Mars WM, Wegner RE, Li Y, Dai C, Nejak K, Liu Y (2002). Disruption of tissue-type plasminogen activator gene in mice reduces renal interstitial fibrosis in obstructive nephropathy. J Clin Invest.

[10] Yang J, Zhang X, Li Y, Liu Y (2003). Downregulation of smad transcriptional corepressors snon and ski in the fibrotic kidney: an amplification mechanism for TGF-beta1 signaling. J Am Soc Nephrol.

[11] Lan HY (2011). Diverse roles of TGF-beta/smads in renal fibrosis and inflammation. Int J Biol Sci.

[12] Meng XM, Chung AC, Lan HY (2013). Role of the TGF-beta/BMP-7/smad pathways in renal diseases. Clin Sci (Lond).

[13] Kalluri R, Neilson EG (2003). Epithelial-mesenchymal transition and its implications for fibrosis. J Clin Invest.

[14] Branton MH, Kopp JB (1999). TGF-beta and fibrosis. Microbes Infect.

[15] Patel V, Noureddine L (2012). MicroRNAs and fibrosis. Curr Opin Nephrol Hypertens.

[16] Bartel DP (2004). MicroRNAs: genomics, biogenesis, mechanism, and function. Cell.

[17] Wienholds E, Kloosterman WP, Miska E, Alvarez-Saavedra E, Berezikov E, de Bruijn E, Horvitz HR, Kauppinen S, Plasterk RH (2005). MicroRNA expression in zebrafish embryonic development. Science.

[18] Yi R, O’Carroll D, Pasolli HA, Zhang Z, Dietrich FS, Tarakhovsky A, Fuchs E (2006). Morphogenesis in skin is governed by discrete sets of differentially expressed microRNAs. Nat Genet.

[19] Esquela-Kerscher A, Slack FJ (2006). Oncomirs - microRNAs with a role in cancer. Nat Rev. Cancer.

[20] Zarjou A, Yang S, Abraham E, Agarwal A, Liu G (2011). Identification of a microRNA signature in renal fibrosis: role of miR-21. Am J Physiol Renal Physiol.

[21] Xiong M, Jiang L, Zhou Y, Qiu W, Fang L, Tan R, Wen P, Yang J (2012). The miR-200 family regulates TGF-beta1-induced renal tubular epithelial to mesenchymal transition through smad pathway by targeting ZEB1 and ZEB2 expression. Am J Physiol Renal Physiol.

[22] Wang B, Komers R, Carew R, Winbanks CE, Xu B, Herman-Edelstein M, Koh P, Thomas M, Jandeleit-Dahm K, Gregorevic P, Cooper ME, Kantharidis P (2012). Suppression of microRNA-29 expression by TGF-beta1 promotes collagen expression and renal fibrosis. J Am Soc Nephrol.

[23] Korpal M, Lee ES, Hu G, Kang Y (2008). The miR-200 family inhibits epithelial-mesenchymal transition and cancer cell migration by direct targeting of E-cadherin transcriptional repressors ZEB1 and ZEB2. J Biol Chem.

[24] Calzado MA, Renner F, Roscic A, Schmitz ML (2007). HIPK2: a versatile switchboard regulating the transcription machinery and cell death. Cell Cycle.

[25] Lee W, rews BC, Faust M, Walldorf U, Verheyen EM (2009). Hipk is an essential protein that promotes notch signal transduction in the drosophila eye by inhibition of the global co-repressor groucho. Dev Biol.

[26] Lee W, Swarup S, Chen J, Ishitani T, Verheyen EM (2009). Homeodomain-interacting protein kinases (Hipks) promote Wnt/Wg signaling through stabilization of beta-catenin/Arm and stimulation of target gene expression. Development.

[27] Zhang J, Pho V, Bonasera SJ, Holtzman J, Tang AT, Hellmuth J, Tang S, Janak PH, Tecott LH, Huang EJ (2007). Essential function of HIPK2 in TGFbeta-dependent survival of midbrain dopamine neurons. Nat Neurosci.

[28] D’Orazi G, Cecchinelli B, Bruno T, Manni I, Higashimoto Y, Saito S, Gostissa M, Coen S, Marchetti A, Del Sal G, Piaggio G, Fanciulli M, Appella E, Soddu S (2002). Homeodomain-interacting protein kinase-2 phosphorylates p53 at ser 46 and mediates apoptosis. Nat Cell Biol.

[29] Hofmann TG, Möller A, Sirma H, Zentgraf H, Taya Y, Dröge W, Will H, Schmitz ML (2002). Regulation of p53 activity by its interaction with homeodomain-interacting protein kinase-2. Nat Cell Biol.

[30] Rinaldo C, Prodosmo A, Siepi F, Soddu S (2007). HIPK2: a multitalented partner for transcription factors in DNA damage response and development. Biochem Cell Biol.

[31] Jin Y, Ratnam K, Chuang PY, Fan Y, Zhong Y, Dai Y, Mazloom AR, Chen EY, D’Agati V, Xiong H, Ross MJ, Chen N, Ma’ayan A, He JC (2012). A systems approach identifies HIPK2 as a key regulator of kidney fibrosis. Nat Med.

[32] Ricci A, Cherubini E, Ulivieri A, Lavra L, Sciacchitano S, Scozzi D, Mancini R, Ciliberto G, Bartolazzi A, Bruno P, Graziano P, Mariotta S (2013). Homeodomain-interacting protein kinase2 in human idiopathic pulmonary fibrosis. J Cell Physiol.

[33] Wang B, Koh P, Winbanks C, Coughlan MT, McClelland A, Watson A, Jandeleit-Dahm K, Burns WC, Thomas MC, Cooper ME, Kantharidis P (2011). miR-200a prevents renal fibrogenesis through repression of TGF-beta2 expression. Diabetes.

[34] Tamagawa S, Beder LB, Hotomi M, Gunduz M, Yata K, Grenman R, Yamanaka N (2014). Role of miR-200c/mir-141 in the regulation of epithelial-mesenchymal transition and migration in head and neck squamous cell carcinoma. Int J Mol Med.

[35] Wallace K, Burt AD, Wright MC (2008). Liver fibrosis. Biochem J.

[36] Wynn TA (2011). Integrating mechanisms of pulmonary fibrosis. J Exp Med.

[37] Yang J, Liu Y (2001). Dissection of key events in tubular epithelial to myofibroblast transition and its implications in renal interstitial fibrosis. Am J Pathol.

[38] Gregory PA, Bert AG, Paterson EL, Barry SC, Tsykin A, Farshid G, Vadas MA, Khew-Goodall Y, Goodall GJ (2008). The mir-200 family and miR-205 regulate epithelial to mesenchymal transition by targeting ZEB1 and SIP1. Nat Cell Biol.

[39] Hill C, Flyvbjerg A, Gronbaek H, Petrik J, Hill DJ, Thomas CR, Sheppard MC, Logan A (2000). The renal expression of transforming growth factor-beta isoforms and their receptors in acute and chronic experimental diabetes in rats. Endocrinology.

[40] Chalazonitis A, Tang AA, Shang Y, Pham TD, Hsieh I, Setlik W, Gershon MD, Huang EJ (2011). Homeodomain interacting protein kinase 2 regulates postnatal development of enteric dopaminergic neurons and glia via BMP signaling. J Neurosci.

[41] Harada J, Kokura K, Kanei-Ishii C, Nomura T, Khan MM, Kim Y, Ishii S (2003). Requirement of the co-repressor homeodomain-interacting protein kinase 2 for ski-mediated inhibition of bone morphogenetic protein-induced transcriptional activation. J Biol Chem.

[42] Hofmann TG, Stollberg N, Schmit ML, Will H (2003). HIPK2 regulates transforming growth factor-beta-induced c-jun NH (2)-terminal kinase activation and apoptosis in human hepatoma cells. Cancer Res.

